# Targeting Bcl-2 stability to sensitize cells harboring oncogenic *ras*

**DOI:** 10.18632/oncotarget.4084

**Published:** 2015-05-25

**Authors:** Bo Peng, Suthakar Ganapathy, Ling Shen, Junchi Huang, Bo Yi, Xiaodong Zhou, Wei Dai, Changyan Chen

**Affiliations:** ^1^ Center for Drug Discovery, Northeastern University, Boston, MA, USA; ^2^ Institute of Clinical Sciences, Sahlgrenska Academy, Gothenburg, Sweden; ^3^ The Jiangxi Province Tumor Hospital, Nanchang, China; ^4^ The First Affiliated Hospital of Nanchang University, Nanchang University School of Medicine, Nanchang, China; ^5^ Department of Environmental Medicine, New York University, Tuxedo, NY, USA

**Keywords:** apoptosis, Bcl-2, protein degradation

## Abstract

The pro-survival factor Bcl-2 and its family members are critical determinants of the threshold of the susceptibility of cells to apoptosis. Studies are shown that cells harboring an oncogenic *ras* were extremely sensitive to the inhibition of protein kinase C (PKC) and Bcl-2 could antagonize this apoptotic process. However, it remains unrevealed how Bcl-2 is being regulated in this apoptotic process. In this study, we investigate the role of Bcl-2 stability in sensitizing the cells harboring oncogenic *K-ras* to apoptosis triggered by PKC inhibitor GO6976. We demonstrated that Bcl-2 in Swiss3T3 cells ectopically expressing or murine lung cancer LKR cells harboring *K-ras* rapidly underwent ubiquitin-dependent proteasome pathway after the treatment of GO6976, accompanied with induction of apoptosis. In this process, Bcl-2 formed the complex with Keap-1 and Cul3. The mutation of serine-17 and deletion of BH-2 or 4 was required for Bcl-2 ubiquitination and degradation, which elevate the signal threshold for the induction of apoptosis in the cells following PKC inhibition. Thus, Bcl-2 appears an attractive target for the induction of apoptosis by PKC inhibition in cancer cells expressing oncogenic *K-ras*.

## INTRODUCTION

Bcl-2 family consists of members that are able either to promote cell survival (such as Bcl-2 and Bcl-XL) or apoptosis (for example, Bax or Bak) [1, 2]. Levels or balances between the pro-and anti-apoptotic factors determine thresholds of cells for the induction of apoptosis. Bcl-2 resides in the endoplasmic reticulum and mitochondria [3, 4]. Through binding to Bax or regulating the membrane permeability of the mitochondria, Bcl-2 was shown to interfere with releasing of the mitochondrial apoptotic factors (for example, Bax or cytochrome c) to the cytosol, and thereby suppressed cell death process [3, 5, 6]. Studies also showed that increases of the expression of Bcl-2 have correlated well with the development of human malignancies, for example, in lung cancer [7–10]. Furthermore, *in vitro* and *in vivo* experiments also demonstrated that a significant change of Bcl-2 in tumorigenesis, inflammatory process, heart failure or other diseases [11–15]. However, the mechanisms by which Bcl- 2 is being regulated and further affects the susceptibility of cells to apoptosis are not fully understood yet.

The protein degradation is a key event in the regulation of various functions or activities of cells [16–18]. The ubiquitination-proteasome pathway is one of the major protein degradation pathways and proven to timingly controls the amount of the expression of proteins involved in the regulation of each critical cellular activity, such as cell cycle checkpoints, tumor surveillances, cellular or DNA damage repair and duration of intracellular signal transduction. In the process of ubiquitination, protein degradation is triggered by E1 ubiquitin activating enzyme. Subsequently, E2 ubiquitin-conjugating enzymes transfer ubiquitin from E1 to E3 to catalyze substrate ubiquitination [19, 20]. Proteins are covalently bound to ubiquitin that is a polypeptide with 76 amino acids and ubiquitously expressed in cells [16–18]. Ubiquitin-tagged proteins eventually are recognized by the proteasome and being degraded, in which lysine residue appears critical [21, 22].

Keap1 is an adaptor protein and participates in Cul3-mediated degradation of Nrf2 during oxidative or radiation-induced stresses [18, 23–25]. Functioning as a sensor for cellular stresses, Keap1 has been shown to be critical for the inhibition of Nrf2 and Bcl-2 activities [26–28]. Keap1 conjugates with the Cul3-containing E3 ubiquitin ligase and mediates Nrf2 and Bcl-2 for their ubiquitination and degradation. The degradation of Bcl-2 in TNF-α or staurosporin-stimulated cells was reported to be through the ubiquitination [29, 30]. Upon the stimulations, Bcl-2 was interacted with Keap1 and then being rapidly degraded, leading to dramatically sensitizing the cells to TNF-α- or staurosporin-induced apoptosis.

Bcl-2 exerts its function through affecting the membrane potential of the mitochondria and activation of caspases [31, 32]. Bcl-2 proteins share 4 sequence homology domains (BH 1–4). Through BH 1–4 domains, Bcl-2 members are able to form homodimers and heterodimers in order to influence the susceptibility of cells to apoptosis. The expression levels of Bcl-2 have been demonstrated to be a crucial factor in cancer progression and development (33, 34). Bcl-2 expression was also proven to play a potential role in inflammatory reactions [11, 35]. Studies indicated that Bcl-2 interacted with Keap1 and further being degraded through Keap1-mediated ubiquitination [26–28]. The interference of Bcl-2 degradation promoted cell survival, suggesting the importance of this protein modulation process in the regulation of cell fate.

This study aimed at getting insights into how Bcl- 2 is being regulated in the cancerous cells expressing oncogenic *K-ras* after the treatment of PKC inhibitor. We demonstrated that Bcl-2 degradation appeared to be involved in apoptosis triggered by GO6976 (an inhibitor specific for PKC α and β) or *shRNA-PKC α* plus *β* in Swiss3T3 cells transformed by *v-K-ras* or murine lung cancer LKR cells harboring oncogenic *K-ras*. In this apoptotic process, Bcl-2 rapidly bound to Keap1 and Cul3 for being ubiquitinated and further degraded. Using *bcl-2* mutants mutated at several lysine sites or deletion mutants at BH 1–4 domains, we showed that the lysine-17 and BH-2 of this pro-survival factor are crucial for the ubiquitination. Our study suggested that Bcl-2 is an attractive target for sensitizing cancer cells expressing oncogenic *K-ras* after being treated with the PKC inhibitor.

## RESULTS

### Bcl-2 is important for sensitizing the cells expressing oncogenic *K-ras* to apoptosis triggered by GO6976

**Figure 1:**
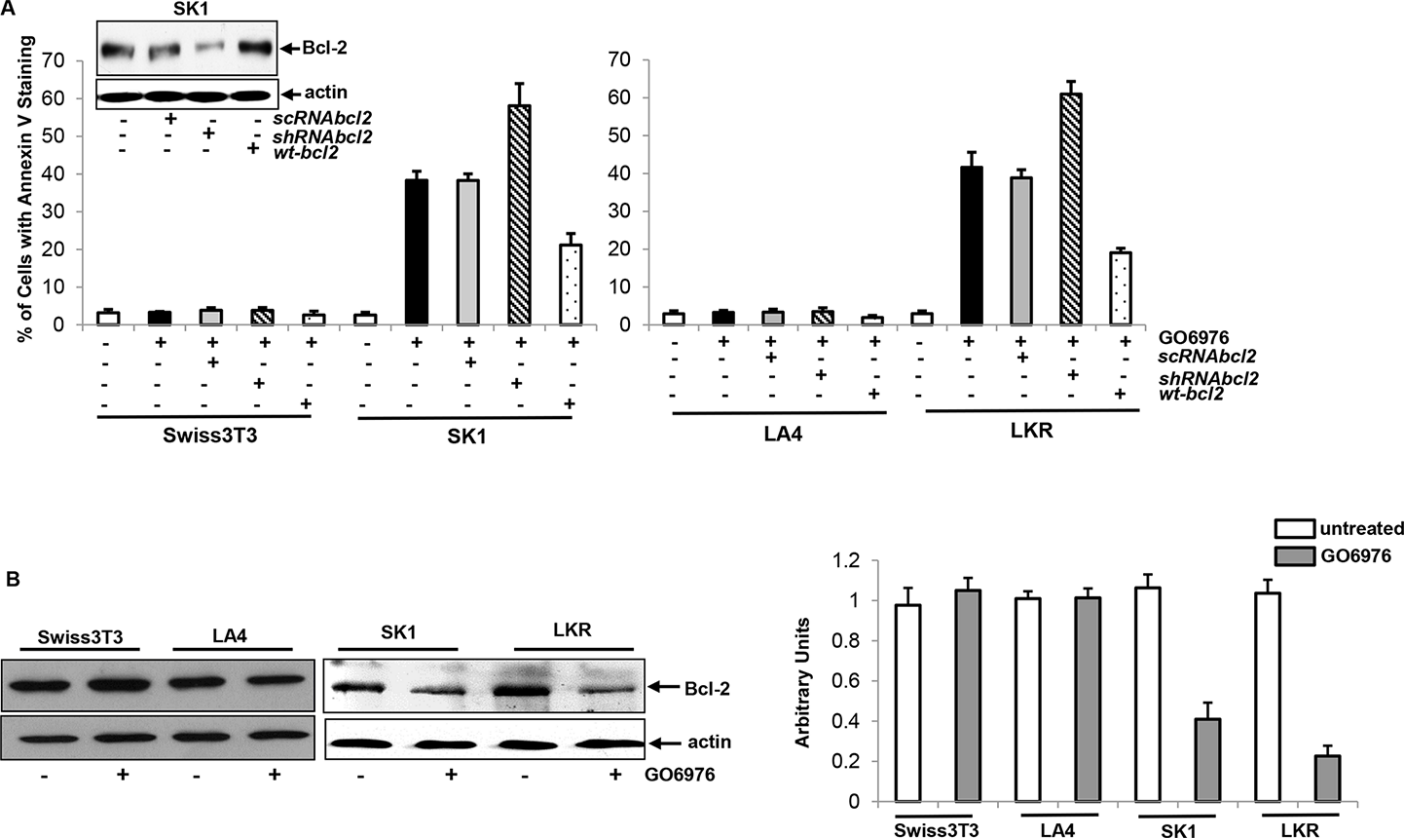
Effect of Bcl-2 on the onset of apoptosis induced by GO6976 **A.** Swiss3T3, SK1, LA4 and LKR cells were transfected with *sc, sh-RNAbcl-2* or *wt-bcl-2*, respectively, prior to the treatment of GO6976 (1 μg/ml) for 48 h. Subsequently, cell lysates were isolated and annexin V assay was performed. The error bars are standard deviation (SD) from 5 independent experiments (*n* = 5, *p* < 0.001). The knockdown or overexpression of *bcl-*2 was analyzed by immunoblotting. The blots were re-probed with anti β-actin antibody to determine equal loading of total proteins per lane. **B.** Cells were treated with GO6976. Cell lysates were prepared and immunoblotting was performed for Bcl-2 expression (left panels). The blots were re-probed with anti β-actin antibody to determine equal loading of total proteins per lane. The expression levels of Bcl-2 in untreated or treated cells were quantitated from 3 independent experiments (right panel, *n* = 3, *p* < 0.005).

Oncogenic Ras is able not only promote cell growth or differentiation, but also function as a pro-apoptotic factor in sensitizing various types of cancer cells under certain circumstances (such as the abrogate of PKC) [36, 37]. To test whether mutated *K-ras* could transmit the apoptotic signaling in our experimental setting, murine fibroblasts Swiss3T3, SK1 (Swiss3T3 cells stably transfected with *v-ras*), murine Lung epithelial LA4 and LKR (that were isolated from the lung neoplasia foci of the *K-ras* transgenic mouse) cells were infected with *sc*, *shRNA-bcl-2*, or *wt-bcl-2*, prior to the treatment of GO6976 (a specific PKC α and β inhibitor) that was proven to induced apoptosis as efficient as co-knockdown by *shRNAα* and *β* under our experimental setting [37]. Subsequently, Annexin V assay was conducted (Fig 1A). After the treatments, only a few of Swiss3T3 or LA4 cells were apoptotic. In comparison, more than 35% of SK1 or LKR cells underwent apoptosis. The overexpression of *wt-bcl-2* moderately suppressed the magnitude of apoptosis in the cells expressing oncogenic *K-ras*, following the addition of GO6976. The knockdown of *bcl-2* significantly increased the susceptibility of SK1 and LKR cells to apoptosis induced by GO6976. The results suggested that Bcl-2 is a rate limiting factor in this apoptotic process.

The correlation between Bcl-2 expression and the resistance to the induction of apoptosis has been reported [38, 39]. In response to growth-related stimulation, Bcl-2 was shown to be upregulated and further desensitize cancer cells to apoptosis [38, 40, 41]. Therefore, the expression of Bcl-2 was examined in the cells after being treated with GO6976 for 48 h when the occurrence of apoptosis became evident (Fig 1B, left panels) and the amounts of the expression were measured (Fig 1B, right panel). Bcl-2 expression in the treated cells were decreased, in comparing with that in untreated cells, which further supported the notion that Bcl-2 is an important factor in this apoptotic process.

### Bcl-2 degradation and ubiquitination were accelerated in the treated cells expressing oncogenic *K-ras*

**Figure 2:**
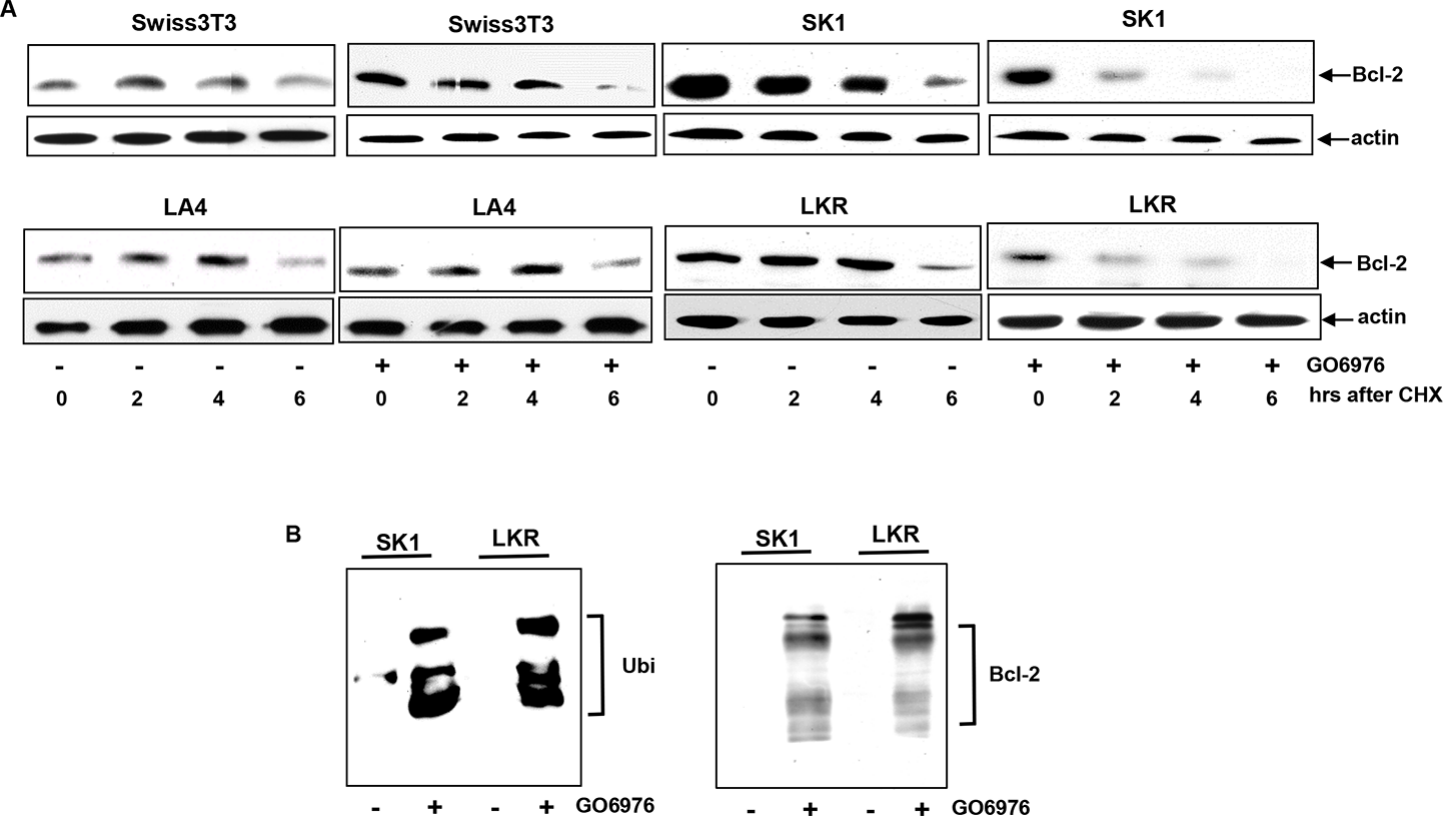
Acceleration of Bcl-2 degradation in the apoptotic process **A.** After the treatment of GO6976, CHX (20 μM) was added into the cell cultures. Lysates were isolated every 2 h and immunoblotting was performed using anti-Bcl-2 antibody. The blot was re-probed with anti β-actin antibody to determine equal loading of total proteins per lane. **B.** SK1 and LKR cells were treated with GO6976 and the cell lysates were prepared. Subsequently, cell lysates were immunoprecipitated with anti-Bcl-2 antibody and the immunoprecipitates were immunobloted with anti-ubiquitin antibody (left panel). The reciprocal co-IP and immunoblotting was performed (left panel).

Bcl-2 is a crucial factor in the regulation of the sensitivity of cell to apoptotic stimulation. The overexpression or knockdown of *bcl-2* changed the response of the cells harboring oncogenic *K-ras* to GO6976 (Fig 1A) and the expression of this pro-survival factor in the cells was also altered upon GO6976 treatment (Fig 1B). It led us to explore the importance of Bcl-2 stability in our experimental setting. RT-PCR (Real-Time PCR) analysis was performed to test whether the change of Bcl-2 expression in this apoptotic process was at the transcriptional level. The kinetics of the gene expression of *bcl-2* in the cells expressing oncogenic *K-ras* with or without GO6976 treatment was similar, suggested that the transcriptional machinery was not involved in the regulation of Bcl-2 expression in this apoptotic process (data not shown). Subsequently, the protein stability of Bcl-2 was examined by protein pulse-chasing analysis (Fig 2A). Cell lysates from GO6976-treated or untreated cells were prepared at various time points after adding CHX to block the synthesis of proteins and then subjected to immunoblotting. The kinetics of the degradation of Bcl-2 in GO6976-treated SK1 and LKR cells was much faster than that in untreated cells. The addition of the PKC inhibitor had no a significant effect on Bcl-2 stability in Swiss3T3 or LA4 cells. Thus, the data indicated that the protein stability of Bcl-2 played a significant role in this apoptotic process in an oncogenic K-Ras-dependent fashion.

Ubiquitination is one of important mechanisms for protein degradation. In this process, lysine residues of proteins are covalently conjugated with ubiquitins, prior to being degraded. Bcl-2 was reported to undergo ubiquitination for its degradation [26–30]. Bcl-2 ubiquitination in SK1 or LKR with or without GO6976 treatment was tested by immunoprecipitation using anti-Bcl-2 antibody and immunoblotting with anti-ubiquitin antibody (Fig 2B, left panel). The reciprocal experiment was also performed (Fig 2B, right panel). Bcl-2 was rapidly conjugated with ubiquitin after the addition of the PKC inhibitor, which was not occurred in the untreated cells. The data further suggested that GO6976 treatment accelerated Bcl-2 degradation, which mitigated the anti-apoptotic activities in the cells expressing oncogenic *K-ras*.

### Lysine-17 is critical for Bcl-2 ubiquitination and degradation

**Figure 3:**
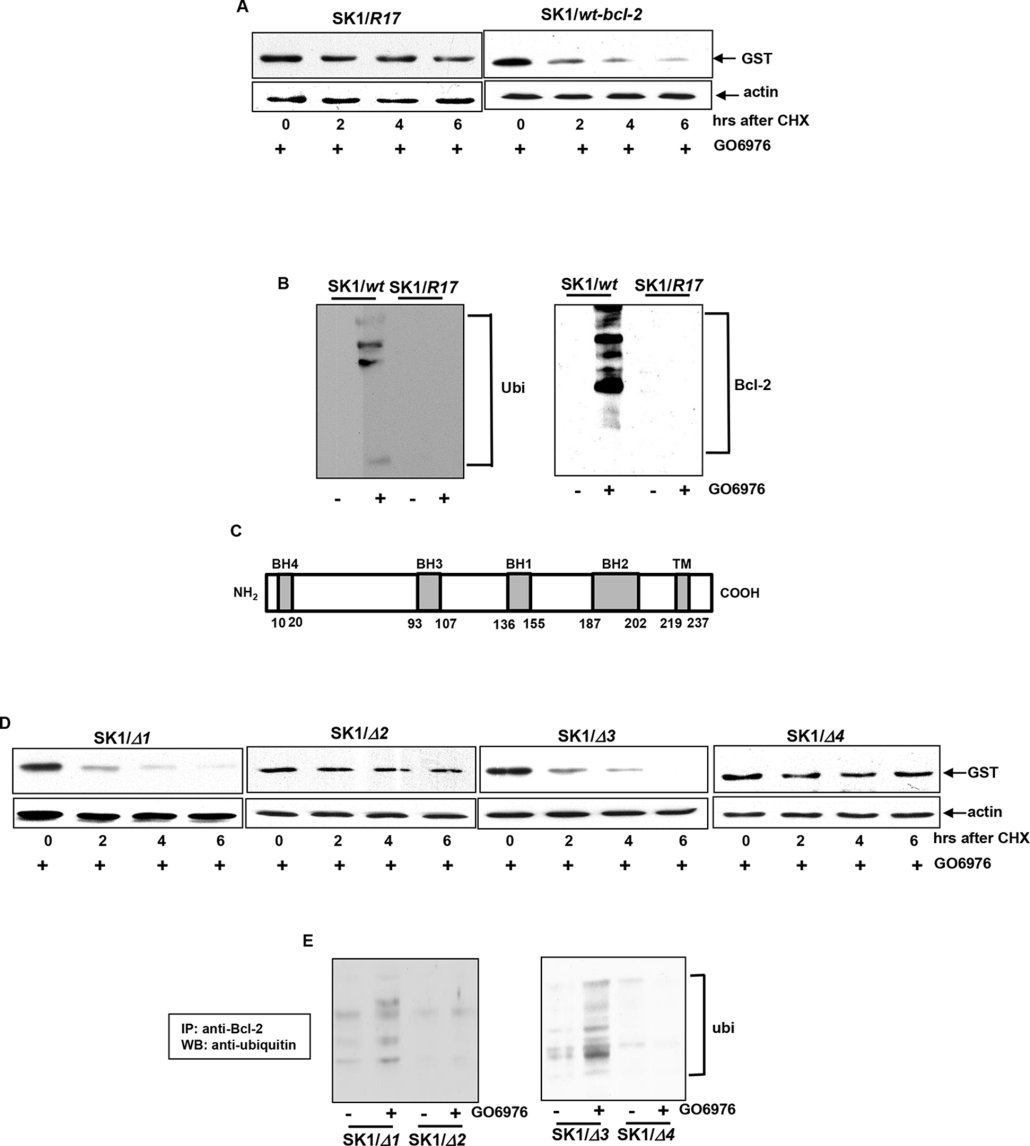
Importance of the lysine 17, BH2 or 4 in Bcl-2 degradation **A.** *bcl-2* mutant with the lysine 17 mutation (R17) or *wt-bcl-2* was constructed in the *pEX-GST* expression vector and then transfected in SK1 cells. After the treatment of GO6976, the degradation of the mutant Bcl-2/R17 or wt-Bcl-2 was examined by immunoblotting using anti-GST antibody. The blot was re-probed with anti β-actin antibody to determine equal loading of total proteins per lane. **B.** Co-IP and immunoblotting of wt-Bcl-2 or Bcl-2/R17 with Ubiquitin in SK1 cells and the reciprocal co-IP/immunoblotting were tested. **C.** Schematic diagram of Bcl-2. The locations of BH domains are presented by grey boxes. **D.** *bcl-2* mutants with various BH deletions in the *pEX-GST* expression vector were transfected into SK1 cells. The transfectants were treated with GO6976 and lystates were prepared. The kinetics of the deletion mutant proteins were tested as described above. **E.** Co-IP and immunoblotting of Bcl-2 deletion mutants and Ubiquitin in SK1 cells and the reciprocal co-IP/immunoblotting were tested.

Bcl-2 undergoes the ubiquitin-proteasomal pathway for its degradation [26–30]. In the first step of this degradation process, lysine residues of proteins are being targeted by the proteasome complexes [21, 22]. The amino acid sequence analysis revealed there are 4 lysine residues existing at the positions of 17, 22, 214 and 236 in Bcl-2 [26, 27]. The residue of lysine 17 was reported to be responsible for Bcl-2 ubiquitination [26, 27]. To test the role of lysine 17 in our experimental setting, *bcl-2* mutant with the mutation of the amino acid 17 to alanine (R17) and *wt-bcl-2* were introduced into SK1 cells. Subsequently, the degradation of Bcl-2/R17 or Bcl- 2 was analyzed in the cells treated with GO6976. The degradation of Bcl-2/R17 was significantly delayed. At 6 h after the addition of cycloheximide (CHX), a moderate amount of Bcl-2 could be detected in GO6976-treated cells (Fig 3A). As expected, wt-Bcl-2 was rapidly degraded in SK1 cells after the treatment. To determine that Bcl-2 degradation was through ubiquitination, the cell lysates were extracted from untreated or treated SK1 cells transfected with *wt-bcl-2* or *bcl-2/R17* vector tagged with *GST*. Immunoprecipitation was performed with anti-GST antibody and immunoblotting with anti-ubiquitin antibody (Fig 3B). The fragmented Bcl-2 conjugated with ubiquitins were present in GO6976-treated SK1/*wt-bcl-2* cells, which was absent in the untreated cells or GO6976-treated SK1/*bcl-2/R17* cells. The result was obtain from the reciprocal experiment. The *bcl-2* mutants at the residue of lysine 22, 214 or 236 that was mutated to alanine were also introduced into the cells under the same experimental condition. The degradation patterns of the protein products of the mutants were similar as that of wt-Bcl-2 (data not shown). The data suggested the lysine-17 of Bcl-2 appears a potential target in regulating the susceptibility of cells harboring oncogenic *ras* to apoptosis in the absence of PKC.

### BH2 or 4 domain of Bcl-2 regulates Bcl-2 stability in GO6976-induced apoptosis

Bcl-2 contains 4 conserved homology domains (BH1–4), each of which interacts with pro- or anti-apoptotic factor, and further positively or negatively regulate apoptosis [42, 43]. The BH1–4 deletion mutants of *bcl-2* were constructed and introduced in SK1 cells, respectively (Fig 3C). After GO6976 treatment, the stability of Bcl-2 with different BH deletions was tested (Fig 3D). The exogenous Bcl-2 with either the deletion of BH1 or 3 was rapidly degraded in response to GO6976 treatment in SK1 cells. Interestingly, not only the BH4 deletion mutant protein in which the critical lysine 17 was excluded, but also the BH2 deletion mutant protein that does not contain lysine appeared stable in the absence of PKC in SK1 cells. To confirm this, the occurrence of ubiquitination of these deletion mutants was also analyzed by co-immunoprecipitation with anti-ubiquitin antibody and immunoblotting with anti-Bcl-2 antibody (Fig 3E). The exogenous Bcl-2 with the BH2 or 4 deletion, but not the BH1 or 3 deletion, was unable to be ubiquitinated in SK1 cells treated with GO6976. The data again suggested that the lysine 17 and BH2 domain of Bcl-2 are important for its ubiquitination and subsequent degradation. The inability of the BH4 deletion mutant to be degraded might be due to the lack of the lysine 17.

### Formation of the ubiquitin complexes is perturbed by *R17*, Δ*2* or Δ*4* mutant of *bcl-2*

**Figure 4:**
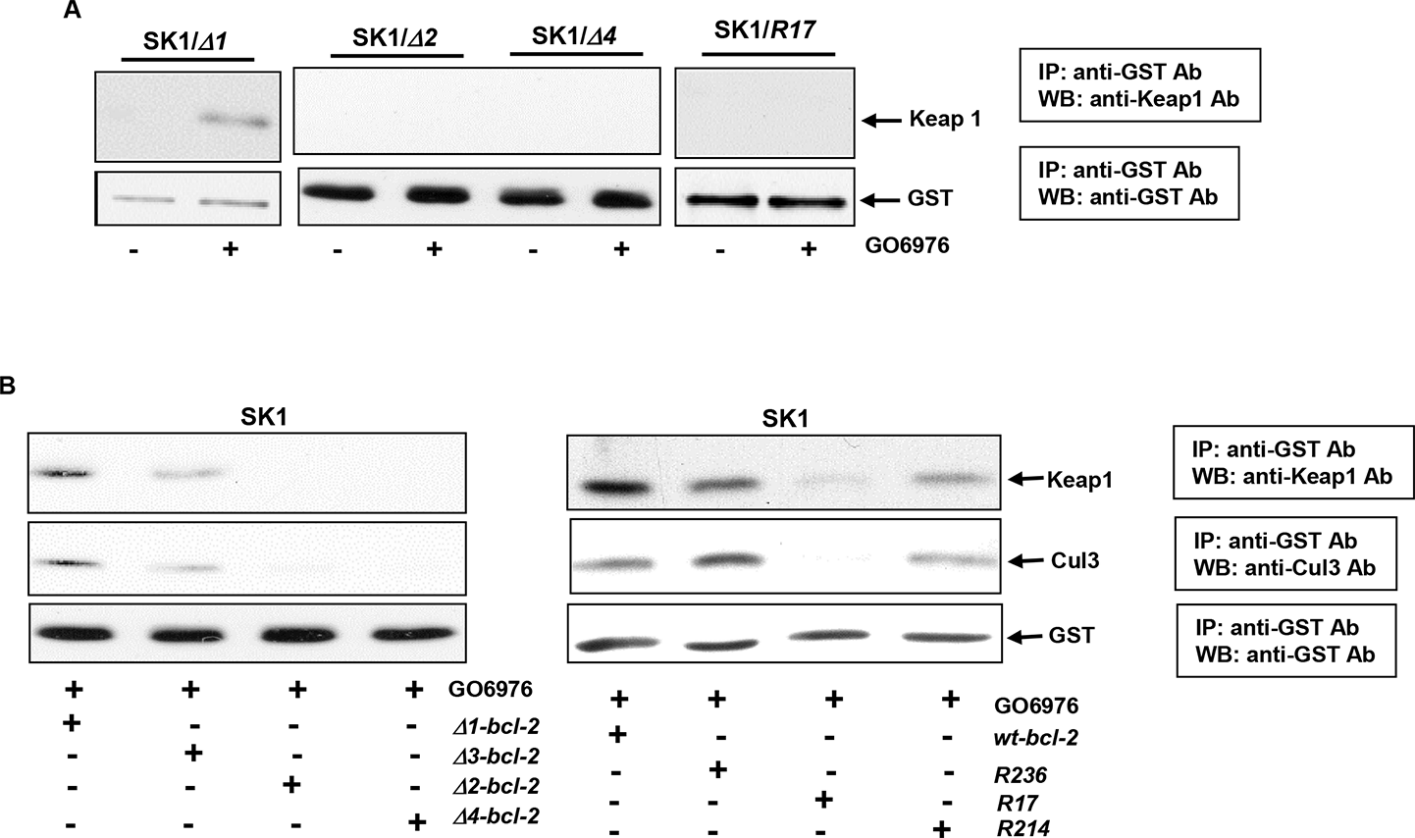
Identification of the interaction domains or residues of Bcl-2 with Keap 1 and Cul3 **A.** SK1 cells expressing different deletion mutants or *bcl-2/R17* mutant were treated with GO6976. Subsequently, cell lysates were immunoprecipitated with anti-GST antibody and immunobloted with an anti-Keap1 antibody. The blot was re-probed with anti-GST antibody for the loading control. **B.** After being transfected with different lysine mutation or BH deletion mutants, cells were immunoprecipitated with anti-GST antibody and immunoblotted with anti-Keap1 or Cul3 antibody. The blots were re-probed with anti β-actin antibody for the determination of equal loading of total proteins per lane.

Keap1, a member of the BTB-Kelch family, serves as an adaptor protein for Cul3-dependent ubiquitination. The interaction of Keap1 with Bcl-2 was shown to promote Bcl-2 ubiquitination and degradation [26, 27]. Therefore, the influences of Bcl-2 mutation and deletion on its association with Keap1 in our experimental setting were tested. After introduced *bcl-2* deletion mutants (Δ*1*, Δ*2* or Δ*4*) or *R17* into SK1 cells, respectively, the co-immunoprecipitation with anti-GST antibody and immunoblotting with anti-Keap1 antibody were performed. The two molecules were bound in GO6976-treated SK1/Δ*1* cells, but not in the cells ectopically expressing Δ*2*, Δ*4* or *R17* (Fig 4A). Next, the existence of Cul3 in Bcl-2/Keap1 complexes was also analyzed in GO6976-treated SK1 cells (Fig 4B). Immunoprecipitation and immumoblotting were conducted in the cells overexpressing *bcl-2* mutants. Consistently, the protein products of the Δ*2*, Δ*4* or *R17* mutants were unable not only to associate with Keap1, but also to bind to Cul3. The data again indicated that the mutation of lysine 17 or deletion of BH4 at N-terminus perturbed the formation of the Cul3 complex for Bcl-2 ubiquitination in SK1 cells treated with GO6976. The results also suggested that BH2 domain was another critical element for the formation of the E3 ligase complexes and, further for promoting Bcl-2 ubiquitination and degradation.

### R17, Δ2 or Δ4 mutant of Bcl-2 desensitizes the cells to apoptosis

**Figure 5:**
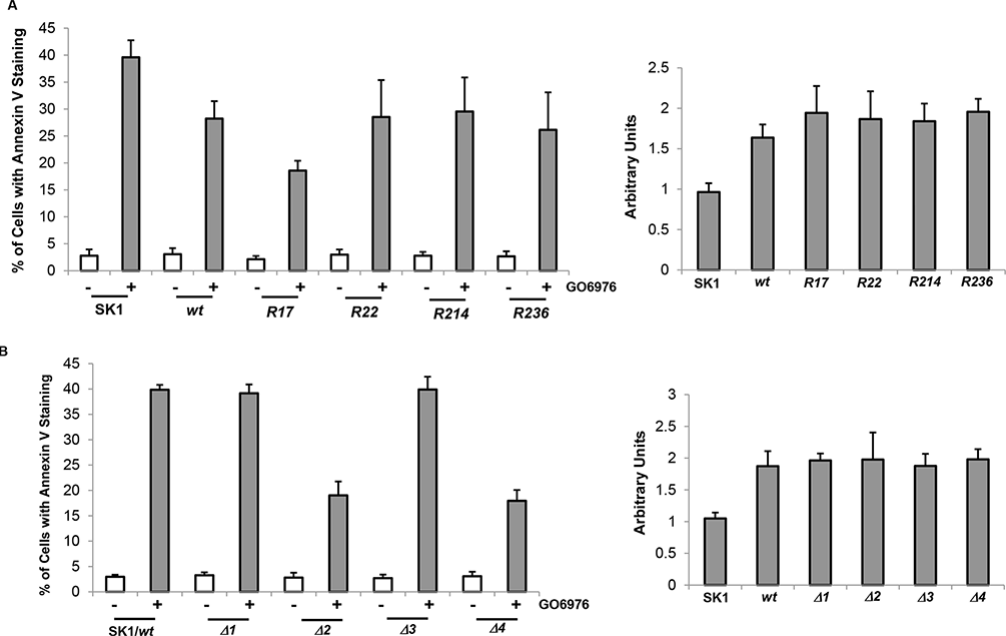
Effect of *bcl-2* mutants on the induction of GO6976-mediated apoptosis SK1 cells were transfected with different lysine mutation *bcl-2* mutants (**A.**, left panel) and BH domain deletion mutants (**B.**, left panel), respectively, prior to the treatment of GO6976 for 48 h. Subsequently, cell lysates were isolated and annexin V assay was performed. The error bars are standard deviation (SD) from 5 independent experiments (*n* = 5, *p* < 0.005). The expression levels of wt- and mu-Bcl-2 were quantitated from 3 independent experiments (right panel, *n* = 3, *p* < 0.005).

To further test the influence of Bcl-2 mutants on the induction of apoptosis mediated by GO6976, Annexin V assay was conducted. After introduced *wt-* and *bcl-*2 mutants, approximately 30% of SK1 cells expressing *bcl-2/R22, R214* or *R236* mutants underwent apoptosis after GO6976 treatment (Fig 5A, left panel). The magnitude of apoptosis in the treated SK1 cells overexpressing these mutants was attenuated in comparison with the treated parental cells, and comparable with the cells overexpressing *wt-bcl-2*. The deletion mutants of *bcl-2* were also transfected into SK1 cells. The ectopic expression levels of wt- or mu-Bcl-2 were also quantitated (Fig 5A, right panel). The magnitude of apoptosis in response to GO6976 treatment was similar in the cells overexpressing *wt-*, Δ*1-* or Δ*3-bcl-2* (Fig 5B, left panel). The amounts of the expression of the deletion mutants were analyzed (Fig 5B, right panel). Consistently, the overexpression of Δ*2-* or Δ*4-bcl-2* significantly reduced the magnitude of GO6976-induced apoptosis in SK1 cells. Together, the data implicated the importance of Bcl-t2 stability in sensitizing SK1 cells to apoptosis after the inhibition of PKC. In this apoptotic process, lysine 17 and BH2 domain appeared critical in the regulation of Bcl-2 degradation.

## DISCUSSION

Active mutations of *ras* can be detected in more than 30% of human malignancies, which has drawn intensive attentions and efforts to understand the structures, biochemistry and biology of this onco-protein, and further to target it as new strategies for treating tumors harboring mutated *ras*. The notion of the therapeutic approaches is that in order to maintain and cope with a high metabolic rate of a cancer cell, oncoproteins need the supports from parallel to or distal of signaling pathways. Disruption of the supporting signaling would perturb adapted homeostasis in cancer cells and further trigger an apoptotic crisis. PKC has been shown to promote tumor growth and cooperate with oncogenic Ras in tumorigenesis [43, 44]. Studies demonstrated that cancer cells harboring oncogenic *ras* were proven to be sensitive to the inhibition of PKC for the induction of apoptosis [36, 45]. In this process, Bcl-2 appeared to play a counteractive role [45, 46]. In this study, we thoroughly investigated the role of Bcl-2 in this apoptotic process. We demonstrated that the cells harboring oncogenic *K-ras* became more sensitive to the PKC inhibitor when *bcl-2* was knocked down and less responsive upon *bcl-2* overexpression. In this apoptotic process, Bcl-2 was strongly ubiquitinated and rapidly degraded, resulting in sensitizing the cells to apoptosis. Our study suggested that PKC cooperates with oncogenic K-Ras to promote or support the survival of cancer cells, probably via increasing Bcl-2 stability.

The ubiquitin-proteolytic mechanism is an important system for the degradation of some proteins in mammalian cells. In dying cells, levels of ubiquitinated proteins were observed to be increased, implicating the connection between protein degradation and apoptotic processes [47, 48]. The function of Bcl-2 was shown to be controlled at the stages of the transcription, posttranscriptional modifications and degradation [42, 43]. In TNFα-mediated apoptosis, Bcl-2 was ubiquitinated and rapidly degraded in human endothelial cells [29]. Furthermore, Bcl-2 became unstable in human lung and kidney cancer cells upon UV-light exposure [26]. Upon nicotine treatment, it was demonstrated that via activating the mitogenic signaling pathways, Bcl-2 ubiquitination and degradation processes were attenuated, which facilitated the establishment of the chemo-resistance in lung cancer cells [28]. Therefore, the proteolytic process regulating Bcl-2 stability appears one of the key players in sensitizing cancer cells expressing oncogenic *ras* to apoptosis.

Keap1 plays a crucial role in regulating the expression of Nfr2, Bcl-2 and other proteins and further cellular activities [23–25]. In antioxidant response, Keap1 was demonstrated to be one of the components in the E3 ubiquitin ligase complex, which controls ubiquitination and proteasomal degradation of the transcriptional factor Nrf2. In TNFα-induced apoptosis, MAPK/ERK1/2 signaling was suggested to be responsible for Bcl- 2 activation [29]. Due to the interaction with Keap1, the negative effect of Bcl-2 on Bax was lifted, which further sensitized cells to apoptosis [26]. The treatment with MAPK inhibitor blocked Bcl-2 phosphorylation and subsequently triggered the degradation of this pro-survival factor. We previously demonstrated that nicotine treatment delayed Bcl-2 dephosphorylation and further increased its stability, which caused Bcl-2 to be unable to associate with Keap1 and delayed to be degraded. In this study using GO6976-induced apoptosis, we further proved the importance of Keap1 in regulating apoptosis involved in Bcl-2. Keap1 appears, through controlling Bcl-2 expression levels, to function as a molecular sensor to regulate apoptosis in cancer cells harboring oncogenic *ras*.

One of the mechanisms by which Bcl-2 exerts its pro-survival function is through its BH (Bcl-2 homology) 1–4 domains [42, 43]. Some apoptotic factors, such as Bax and Bik, also possess BH domains. Through BH domains, Bcl-2 associates with pro-apoptotic factors and regulates the fate of a cell. The stability of Bcl-2 was shown to influence the susceptibility of cells to apoptosis [29]. Upon the exposure of UV light, the lysine 17 was demonstrated to be critical for the ubiquitination of this pro-survival factor [26]. In our experimental setting, Bcl-2, once its lysine 17 was mutated or deleted, was unable to be degraded in the cells expressing oncogenic *ras* after PKC suppression. Interestingly, Bcl-2 became very stable when its BH2 domain that does not contain lysine residues, was deleted. It is possible the BH2 domain serves to anchor one of the components of Cul3-containing E3 ubiquitin ligase and is necessary for Bcl-2 degradation. The further investigation of the role of the BH2 domain of Bcl-2 is under way.

In summary, we have demonstrated that Bcl-2 ubiquitination and degradation play an important role in sensitizing cancer or transformed cells expressing oncogenic *ras* to apoptosis triggered by PKC inhibition. Our study also identified that impairment of Bcl-2 by mutating its lysine 17 or deleting BH2 domain prolonged the half-life of this pro-survival factor and further desensitized the cells to apoptosis induced by GO6976. Thus, Bcl-2 dictates cell viability and death not only through its heterodimerization with apoptotic factors, but also being controlled by Keap1/Cul3-mediated ubiquitination. Dysregulation of Keap1 or Cul3 function would definitely affect cell survival involving Bcl-2.

## MATERIALS AND METHODS

### Cell lines and treatments

Swiss3T3 and LA4 cells were obtained from ATCC (Manassas, VA, USA). LKR murine lung cells are generous gift from Dr. Jacks (MIT, Cambridge) and have been used in various studies [49–51]. The cells were cultured in DMEM medium containing 10% fetal calf serum. SK1 cells are Swiss3T3 cells stably transfected with *v-K-ras* and maintained in the growth medium containing G418 (200 μg/ml). GO6976 was purchased from Sigma. *Bcl-2* mutants were inserted in the *pEX-GST* expression vector and obtained from Dr. Luo (Boston University).

### Annexin V-FITC apoptosis detection assay

After treatments, cells were harvested and stained with Annexin V-FITC Apoptosis Detection Kit I (BD Biosciences) according to manufacturer's instructions and subsequently, analyzed by a flow cytometer.

### Immunoprecipitation and immunoblotting analyses

For immunoprecipitation, after treatments, cell lysates were isolated and immunoprecipitated with an antibody. The precipitates were then separated on a SDS-PAGE gel for immunoblotting (see below).

For immunoblotting, lysates were isolated from treated cells and separated by SDS-PAGE gels. Following the transfer, nitrocelluloses were incubated with the designated primary antibodies overnight at 4°C, and subsequently incubated with second antibodies for 2 h at room temperature. The blots were finally detected by chemiluminescence.

### Statistical analysis

Three to five independent repeats were conducted in all experiments. Error bars represent these repeats. A Student's *T* test was used and a *p* value of < 0.05 was considered significant.

## References

[1] García-Sáez AJ (2012). The secrets of the Bcl-2 family. Cell Death Differ.

[2] Yip KW, Reed JC (2008). Bcl-2 family proteins and cancer. Oncogene.

[3] Kroemer G (1997). The proto-oncogene Bcl-2 and its role in regulating apoptosis. Nat. Med.

[4] Krajewski S, Tanaka S, Takayama S, Schibler MJ, Fenton W, Reed JC (1993). Investigation of the subcellular distribution of the bcl-2 oncoprotein: residence in the nuclear envelope, endoplasmic reticulum and outer mitochondrial membranes. Cancer Res.

[5] Kluck RM, Bossy-Wetzel E, Green DR, Newmeyer DD (1997). The release of cytochrome c from mitochondria: a primary site for Bcl-2 regulation of apoptosis. Science.

[6] Rong Y, Distelhorst CW (2008). Bcl-2 protein family members versatile regulators of calcium signaling in cell survival and apoptosis. Annu Rev Physiol.

[7] Gallo O, Bianchi S, Porfirio B (1995). Bcl-2 overexpression and smoking history in head and neck cancer. J Natl Cancer Inst.

[8] Borner MM, Brousset P, Pfanner-Meyer B, Bacchi M, Vonlanthen S, Hotz MA, Altermatt HJ, Schlaifer D, Reed JC, Betticher DC (2002). Expression of apoptosis regulatory proteins of the Bcl-2 family and p53 in primary resected non-small-cell lung cancer. Br J Cancer.

[9] Zereu M, Vinholes JJF, Zettler GC (2003). p53 and Bcl-2 protein expression and its relationship with prognosis in small cell lung cancer. Clin Lung Cancer.

[10] Assis GF, Ceolin DS, Marques ME, Salvadori DM, Ribeiro DA (2005). Cigarette smoke affects apoptosis in rat tongue mucosa: role of bcl-2 gene family. J Mole Histol.

[11] Wang W, Bergh A, Damber J-E (2004). Chronic inflammation in benign prostate hyperplasia is associated with focal upregulation of cyclooxygenase-2, Bcl-2 and cell proliferation in the glandular epithelium. The Prostate.

[12] Gerber H-P, Dixit V, Ferrara N (1998). Vascular endothelial growth factor induces expression of the antiapoptotic proteins Bcl-2 and A1 in vascular endothelial cells. J. Biol. Chem.

[13] Cory S, Adams JM (2005). Killing cancer cells by flipping the Bcl-2/Bax switch. Cancer Cell.

[14] Adams JM, Cory S (2007). The Bcl-2 apoptotic switch in cancer development and therapy. Oncogene.

[15] Thomas S, Quinn BA, Das SK, Dash R, Emdad L, Dasgupta S, Wang XY, Dent P, Reed JC, Pellecchia M, Sarkar D, Fisher PB (2013). Targeting the Bcl-2 family for cancer therapy. Expert Opin Ther Targets.

[16] Ciechanover A (1994). The ubiquitin-proteasome proteolytic pathway. Cell.

[17] Hochstrasser M (1996). Protein degradation or regulation: Ub the judge. Cell.

[18] Weissman AM (1997). Regulating protein degradation by ubiquitination. Immunol Today.

[19] Hershko A, Ciechanover A (1998). The ubiquitin system. Annu. Rev. Biochem.

[20] Pickart CM (2001). Mechanisms underlying ubiquitination. Annu. Rev. Biochem.

[21] Weissman AM (2001). Themes and variations on ubiquitylation. Nat. Rev. Mole. Cell Bio.

[22] Peng J, Schwartz D, Elias JE, Thoreen CC, Cheng D, Marsischky G, Roelofs J, Finley D, Gygi SP (2003). A proteomics approach to understand protein ubiquitination. Nat. Biotech.

[23] Cullinan SB, Gordan JD, Jin J, Haper JW, Diehl JA (2004). The Keap1BTB protein is an adaptor that bridges Nrf2 to a Cul3-based E3 ligase: oxidative stress sensing by a Cul3-Keap1 ligase. Mole Cell Biol.

[24] Zhang DD, Lo SC, Cross JV, Templeton DJ, Hannink M (2004). Keap1 is a redox regulated substrate adaptor protein for a Cul3-dependent ubiquitin ligase complex. Mole Cell Biol.

[25] Sun Z, Zhang S, Chan JY, Zhang DD (2007). Keap1 controls postinduction repression of the Nrf2-mediated antioxidant response by escorting nuclear export of Nrf2. Mole. Cell. Biol.

[26] Niture SK, Jaiswal AK (2011). INrf2 (Keap1) targets Bcl-2 degradation and controls cellular apoptosis. Cell Death & Diff.

[27] Niture SK, Jaiswal AK Nrf2 protein up-regulates antiapoptotic protein Bcl-2 and prevents cellular apoptosis. J. Biol. Chem.

[28] Nishioka T, Luo L-Y, Shen L, He H, Chen C (2014). (Nicotine increases the resistance of lung cancer cells to cisplatin through enhancing Bcl-2 stability. Bri J of Cancer.

[29] Dimmeler S, Breitschopf K, Kaendeler J, Zeiher AM (1999). Dephosphorylation targets Bcl-2 for ubiqutin-dependent degradation: a link between the apoptosome and the proteasome pathway. J. Exp. Med.

[30] Breitschopf K, Haendeler J, Maichow P, Zeiher AM, Dimmeier S (2000). Posttranlational modification of Bcl-2 facilitates its proteasome-dependent degradation: molecular characterization of the involved signaling pathway. Mole. Cell. Biol.

[31] Youle RJ, Strasser A (2008). The Bcl-2 protein family: opposing activities that mediate cell death. Nat. Rev. Mole. Cell Biol.

[32] Czabotar PE, Lessene G, Strasser A, Adams JM (2014). Control of apoptosis by the Bcl-2 protein family: implications for physiology and therapy. Nat. Rev. Mole. Cell Biol.

[33] Hockenbery DM (1994). Bcl-2 in cancer, development and apoptosis. J. Cell Sci.

[34] Chao DT, Korsmeyer SJ (1998). Bcl-2 family: regulators of cell death. Annu. Rev. Immonol.

[35] Lagasse E, Weissman IL (1994). Bcl-2 inhibits apoptosis of neutrophils but not their engulfment by macrophages. J. Exp. Med.

[36] Ma P, Magut M, Chen X, Chen C p53 is necessary for the apoptotic response mediated by a transient increase of Ras activity. Mole. Cell. Biol.

[37] Zhu T, Tsuji T, Chen C (2010). Roles of PKC isoforms in the induction of apoptosis elicited by aberrant Ras. Oncogene.

[38] Miyashita T, Reed JC Bcl-2 oncoprotein blocks chemotherapy-induced apoptosis in a human cell line. Blood.

[39] Kang MH, Reynolds CP (2009). Bcl-2 inhibitors: targeting mitochondrial apoptotic pathways in cancer therapy. Clin. Cancer Res.

[40] Nishioka T, Kim Y, Luo L-Y, Huang Y, Guo J, Chen C (2011). Sensitization of epithelial growth factor receptor by nicotine exposure to promote breast cancer cell growth. Breast Cancer Res.

[41] Rong Y, Distelhorst CW (2008). Bcl-2 protein family members: versatile regulators of calcium signaling in cell survival and apoptsis. Annu. Rev. Physiol.

[42] Breitschopf K, Haendeler J, Malchow P, Zeiher AM, Dimmeler D (2000). Posttranslational modification of Bcl-2 facilitates its proteasome-dependent degradation: molecular characterization of the involved signaling pathways. Mole. Cell. Biol.

[43] Borner C, Guadagno SN, Hsiao WW, Fabbro D, Barr M, Weinstein IB (1992). Expression of four protein kinase C isoform in rat fibroblasts. J. Biol. Chem.

[44] Kazi JU, Soh J-W (2008). Induction of the nuclear proto-oncogene c-fos by the phorbol ester TPA and c-H-Ras. Mole. Cells.

[45] Chen C, Faller DV (1995). Direction of p21Ras-generated signals towards cell growth or apoptosis is determined by protein kinase C and Bcl-2. Oncogene.

[46] Chen C, Faller DV (1996). Phosphorylation of Bcl-2 protein and association with p21Ras in Ras-induced apoptosis. J. Biol. Chem.

[47] Schwartz LM, Myer A, Kosz L, Engelstein M, Maier C (1990). Activation of polyubiquitin gene expression during developmentally programmed cell death. Neuron.

[48] Orlowski RZ (1999). The role of the ubiquitin-proteasome pathway in apoptosis. Cell Death Differ.

[49] Johnson L, Mercer K, Greenbaum D, Bronson RT, Crowley D, Tuveson DA, Jacks T (2001). Somatic activation of the K-ras oncogene causes early onset lung cancer in mice. Nature.

[50] Matsumura K, Opiekun M, Oka H, Vachani A, Albelda SM, Yamazaki K, Beauchamp GK (2010). Urinary volatile compounds as biomarkers for lung cancer: a proof of principle study using odor signatures in mouse models of lung cancer. Plos One.

[51] Nishioka T, Guo J, Yamamoto D, Chen L, Huppi P, Chen C (2010). Nicotine, through upregulating pro-survival signaling, cooperates with NNK to promote transformation. J of Cell Biochem.

